# Mitochondrial genome of *Hypoderaeum conoideum* – comparison with selected trematodes

**DOI:** 10.1186/s13071-015-0720-x

**Published:** 2015-02-12

**Authors:** Xin Yang, Robin B Gasser, Anson V Koehler, Lixia Wang, Kaixiang Zhu, Lu Chen, Hanli Feng, Min Hu, Rui Fang

**Affiliations:** State Key Laboratory of Agricultural Microbiology, College of Veterinary Medicine, Huazhong Agricultural University, Wuhan, 430070 Hubei PR China; Faculty of Veterinary and Agricultural Sciences, The University of Melbourne, Parkville, 3010 VIC Australia; Hubei Provincial Center for Diseases Control and Prevention, Wuhan, 430079 Hubei PR China; Hubei Entry-Exit Inspection and Quarantine Bureau, Wuhan, 430022 Hubei PR China

**Keywords:** *Hypoderaeum conoideum*, Mitochondrial genome

## Abstract

**Background:**

*Hypoderaeum conoideum* is a neglected but important trematode. The life cycle of this parasite is complex: snails serve as the first intermediate hosts: bivalves, fishes or tadpoles serve as the second intermediate hosts, and poultry (such as chickens and ducks) act as definitive hosts. In recent years, *H. conoideum* has caused significant economic losses to the poultry industry in some Asian countries. Despite its importance, little is known about the molecular ecology and population genetics of this parasite. Knowledge of mitochondrial (mt) genome of *H. conoideum* can provide a foundation for phylogenetic studies as well as epidemiological investigations.

**Methods:**

The entire mt genome of *H. conoideum* was amplified in five overlapping fragments by PCR and sequenced, annotated and compared with mt genomes of selected trematodes. A phylogenetic analysis of concatenated mt amino acid sequence data for *H. conoideum,* eight other digeneans (*Clonorchis sinensis*, *Fasciola gigantica*, *F. hepatica*, *Opisthorchis felineus*, *Schistosoma haematobium*, *S. japonicum*, *S. mekongi* and *S. spindale*) and one tapeworm (*Taenia solium*; outgroup) was conducted to assess their relationships.

**Results:**

The complete mt genome of *H. conoideum* is 14,180 bp in length, and contains 12 protein-coding genes, 22 transfer RNA genes, two ribosomal RNA genes and one non-coding region (NCR). The gene arrangement is the same as in *Fasciola* spp, with all genes being transcribed in the same direction. The phylogenetic analysis showed that *H. conoideum* had a relatively close relationship with *F. hepatica* and other members of the Fasciolidae, followed by the Opisthorchiidae, and then the Schistosomatidae.

**Conclusions:**

The mt genome of *H. conoideum* should be useful as a resource for comparative mt genomic studies of trematodes and for DNA markers for systematic, population genetic and epidemiological studies of *H. conoideum* and congeners.

## Background

Echinostomatid trematodes comprise a group of at least 60 species [1], some of which are of socioeconomic significance in animals. *Hypoderaeum conoideum* (Bloch, 1782) is an important member of the family. This echinostomatid was originally found in the intestines of birds and is known to infect chickens, ducks and geese in many countries around the world [2-4]. It has also been found to infect humans and cause echinostomiasis in Thailand [5,6]. Freshwater snails, *Planorbis corneus*, *Indoplanorbis exustus*, *Lymnaea stagnalis*, *L. limosa*, *L. ovata* and *L. rubiginosa*, act as first intermediate hosts and shed the cercariae; bivalves, fishes or tadpoles can act as second intermediate hosts [3,5].

The accurate identification of species and genetic variants of *Hypoderaeum conoideum* will be central to investigating its biology, epidemiology and ecology, and also has implications for the diagnosis of infections. Although morphological features are used to identify this and other trematodes, such characters are not always reliable [7]. Due to these constraints, various molecular methods have been established for specific identification [7]. For instance, PCR-based techniques using genetic markers in nuclear ribosomal (r) and mitochondrial (mt) DNA have been widely used [7]. The sequences of the first and second internal transcribed spacers (ITS-1 and ITS-2 = ITS) of nuclear rDNA have been particularly useful for specific identification, based on consistent levels of sequence difference between species and little variation within individual species [7], while the mitochondrial gene *cox*1 has been used for studying genetic variation and relationships among different species [8-10]. As a basis for the development of molecular tools to study *H. conoideum* populations (irrespective of developmental stage), we have characterized the complete mt genome of this parasite, compared this genome with those of selected trematodes and undertaken a phylogenetic analysis of concatenated amino acid sequence data for 12 protein-coding genes to assess the genetic relationship of *H. conoideum* with these other trematodes.

## Methods

### Parasites and DNA isolation

*H. conoideum* adults were collected from the intestine of a naturally infected free-range duck in Hubei province, China, in accordance with the Animal Ethics Procedures and Guidelines of Huazhong Agricultural University. These worms were washed in physiological saline and identified morphologically according to existing morphological descriptions [11]. A reference specimen was stained and mounted [12] and the remaining specimens were fixed in 70% (v/v) ethanol and stored at −20°C until use [8]. Total genomic DNA was extracted from one specimen using E.Z.N.A.® Tissue DNA Kit. To provide further identification for this specimen, the ITS-2 region was amplified and sequenced [13], it was identical to a reference sequence available for *H. conoideum* (GenBank accession no. KJ 944311.1).

### Amplification and sequencing of partial *cox*1*, cox*3*, nad*4*, nad*5 and *rrn*S

Initially, ten oligonucleotide primers (Table 1) were designed to regions of the mt genome of *Fasciola hepatica* [14], in order to amplify short fragments from the *cox*1*, cox*3*, nad*4*, nad*5 and the small subunit of ribosomal RNA (*rrn*S) genes (Table 1). PCR (25 μl) was performed in 10 mM Tris–HCl (pH 8.4), 50 mM KCl, 4 mM MgCl_2_, 200 mM each of dNTP, 50 pmol of each primer, 2 U *Taq* polymerase (Takara) and 2.5 μl genomic DNA or H_2_O (no-DNA control) in a thermocycler (Biometra) under the following conditions: an initial denaturation at 94°C for 5 min, followed by 30 cycles of 94°C/1 min; 47–50°C/30 s (depending on primer pair), 72°C/1 min, followed by a final extension of 72°C/7 min. Amplicons were sent to Sangon Company (Shanghai, China) for sequencing by using the same forward and reverse primers (separately) as used in PCR.

**Table 1:** **Sequences of primers used to amplify fragments from** ***Hypoderaeum conoideum***

**Primer codes**	**Sequences(5′ to 3′)**	**Target gene**
XCCOX3F2	AGYACDGTDGGDTTRCATTT	*cox*3^1^
XCCOX3R1	CANAYATAATCMACARAATGNCA	*cox*3^1^
XcND4F	GADTCBCCDTATTCDGARCG	*nad*4^1^
XcND4R	GCHARCCADCGCTTVCCNTC	*nad*4^1^
TXCCOX1F	GGHTGAACHRTWTAYCCHCC	*cox*1^1^
TXCCOX1R	TGRTGRGCYCAWACDAYAMAHCC	*cox*1^1^
Insect12SF	AAWAAYGAGAGYGACGGGCG	*rrn*S^1^
Insect12SR	TARACTAGGATTAGATACCC	*rrn*S^1^
XcND5F	ATGCGNGCYCCNACNCCNGTDAG	*nad*5^1^
XcND5R1	TGCTTVSWAAAAAANACHCC	*nad*5^1^
XCF2	TATTAGGAGGTTTGGTGG	*cox*3*-nad*4^2^
XCR3	ATCATAACTACCACATACCCC	*cox*3*-nad*4^2^
XCF4	TAGGTATTGCTTGTTAGCTG	*nad*4*-cox*1^2^
XCR2	TTTAATCGAACCAAGGACAC	*nad*4*-cox*1^2^
XCF3	CATTAGTCACATTTGTATGAC	*cox*1*- rrn*S^2^
XCR10	GGACTATCTTTTATGATACACG	*cox*1*- rrn*S^2^
XCF1	GTTATTGGGTTTAGGACTCGG	*rrn*S *- nad*5^2^
XCR8	ACTAACACCGTATTCAACTC	*rrn*S *- nad*5^2^
XCF9	TTTCTCTTTGTGGTTTGCCG	*nad*5*-cox*3^2^
XCR1	TATTAGGTTGTGGTACCCC	*nad*5*-cox*3^2^

Primer pairs (top to bottom) used to amplify fragments; ^1^short regions amplified by PCR from *cox*1 (494 bp), *cox*3 (140 bp), *nad*4 (440 bp), *nad*5 (529 bp) and *rrn*S (383 bp). ^2^large fragments that were amplified by long-range PCR from *cox*3-*nad*4 (2048 bp), *nad*4-*cox*1 (4664 bp), *cox*1-*rrn*S (2352 bp), *rrn*S-*nad*5 (2272 bp) and *nad*5-*cox*3 (1752 bp).

### Long-PCR amplification and sequencing

Ten additional primers (see Table 1) were then designed from the sequences obtained, and used to amplify genomic DNA (~40-80 ng) from five regions (see Table 1) by long-PCR; PCRs (25 μl) were performed in a reaction buffer containing 2 mM MgCl_2,_ 1× LA Taq Buffer II, 0.4 mM dNTP mixture, 0.8 μM of each primer, 2.5 U LA *Taq* polymerase (Takara) and 2.5 μl of genomic DNA or H_2_O (no-DNA control) for 35 cycles of 94°C/30 s (denaturation), 50°C/30 s (annealing) and 72°C/1 min (extension) per kb. Amplicons were cloned into pGEM-T-Easy vector (Promega, USA) according to the manufacturer’s protocol; inserts were amplified by long-range PCR (employing vector primers M13 and M14) and then sequenced using a primer-walking strategy [15].

### Sequence analyses

Sequences were assembled using the software ContigExpress program (Invitrogen, Carlsbad, CA), and aligned against the mt genome sequences of other available trematodes (including *F. hepatica*) using the programs Clustal X v.1.83 [16] to infer gene boundaries. The open reading frames (ORFs) were identified using ORF Finder (http://www.ncbi.nlm.nih.gov/gorf/gorf.html) employing the flatworm mitochondrial genetic code. Translation initiation and termination codons were identified as described previously [14,17,18]. The secondary structures of the 22 tRNA genes were predicted using tRNAscan-SE and/or manual adjustment [9,19]. The two rRNA genes were identified by comparison with those from the mt genome of *F. hepatica* [14]. Amino acid sequences of the protein-coding genes were obtained by using the flatworm mt code, and aligned using the program MUSCLE [20] employing default settings.

### Sliding window analysis of nucleotide variation

Sequence variability between *H. conoideum* and *F. hepatica* was conducted by sliding window analysis using the software DnaSP v.5 [21]. A sliding window analyses was implemented as described previously [22].

### Phylogenetic analysis

Amino acid sequences conceptually translated from individual genes of the mt genome of *H. conoideum* were concatenated and aligned with those from available mt genomes of trematodes, including *Clonorchis sinensis* (NC_012147) [14,23], *Fasciola gigantica* (NC_024025) [22], *F. hepatica* (NC_002546) [14], *Opisthorchis felineus* (NC_011127) [23], *Schistosoma haematobium* (NC_008074) [24], *Schistosoma japonicum* (AF215860) [14], *Schistosoma mekongi* (NC_002529) [18], *Schistosoma spindale* (NC_008067) [24], and the cestode *Taenia solium* (outgroup) (NC_004022.1) [25]. The phylogenetic analysis was conducted using the neighbour-joining (NJ) method employing the Tamura-Nei model [20]. Confidence limits were assessed using bootstrap procedure with 1000 pseudo-replicates for neighbour-joining tree, and other settings were obtained using the default values in MEGA v.6.0 [20]. In addition, maximum parsimony (MP), Bayesian (MB) and maximum likelihood (ML) analyses were implemented as described previously by other workers [20,26,27].

## Results

### Features of the mt genome of *H. conoideum*

**Figure 1:**
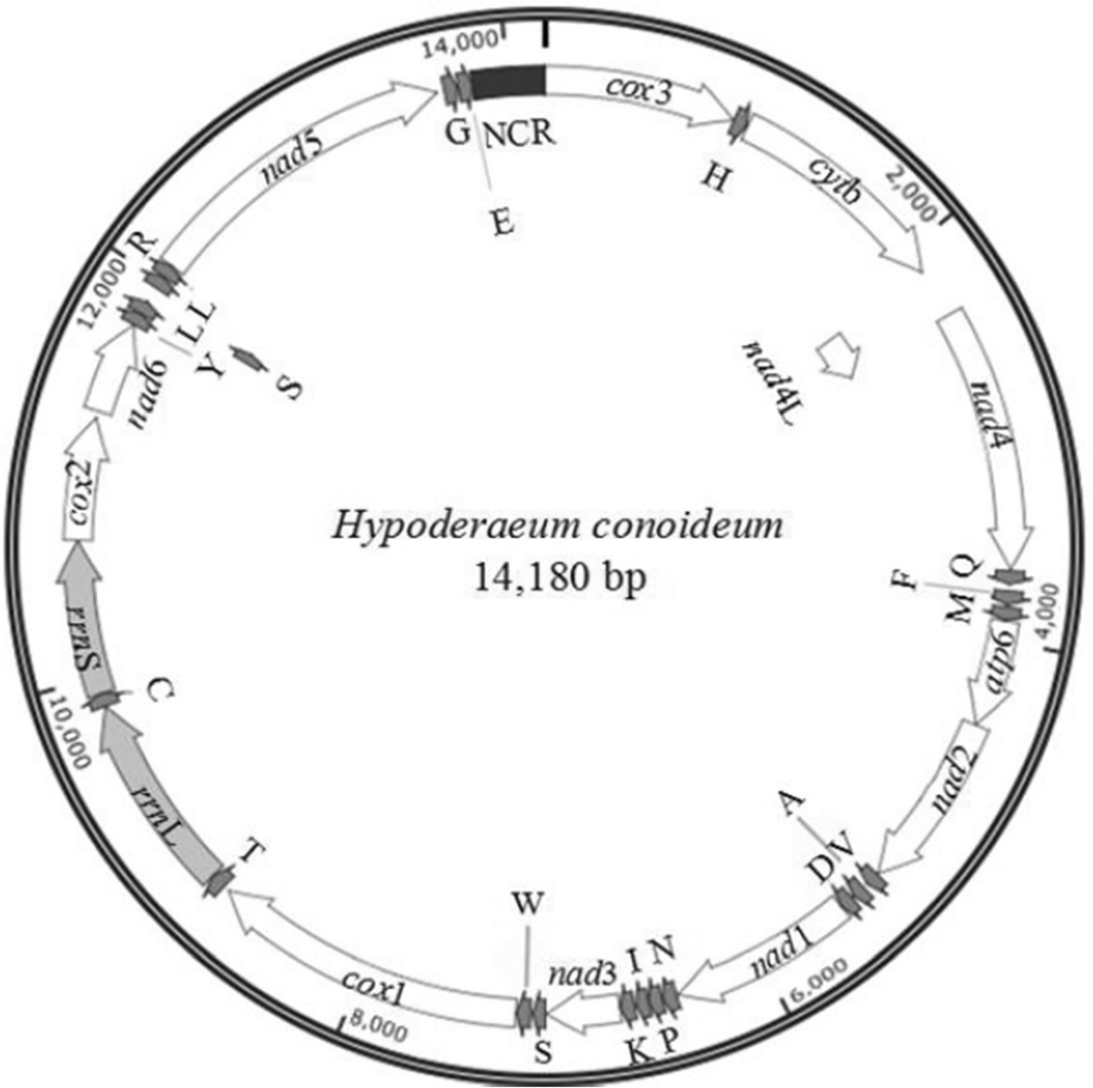
**Organisation of genes in the mitochondrial genome of** ***Hypoderaeum conoideum.***

The circular mt genome of *H. conoideum* (GeneBank accession no. KM_111525) is 14,180 bp in size. It includes 22 tRNA genes, two rRNA genes (*rrn*S and *rrn*L), 12 protein-coding genes (*cox*1-3, *nad*1-6, *nad*4L, *cyt*b and *atp*6) and a non-coding region, but lacks an *atp*8 gene, and all genes are transcribed in the same direction (Figure 1), which is consistent with other trematodes, such as *F. hepatica* [14], *O. felineus* [22] and *S. haematobium* [24]. The arrangement of the protein-encoding genes is: *cox*3-*cyt*b-*nad*4L-*nad*4-*atp*6-*nad*2-*nad*1-*nad*3-*cox*1-*cox*2-*nad*6-*nad*5, which is in accordance with *F. hepatica* [14], *O. felineus* [22], *S. japonicum* [14] and *S. mekongi* [18]*,* but different from that of *S. haematobium* and *S. spindale* [24].

Overlapping nucleotides between the mt genes of *H. conoideum* ranged from 1 to 40 bp (Table 2), which is the same as other for trematodes, such as *F. hepatica* [14] and *O. felineus* [22]. The mt genome of *H. conoideum* has 26 intergenic spacers, each ranging from 1 to 34 bp in length (Table 2). The nucleotide contents in the mt genome are: 18.92% (A), 11.71% (C), 42.46% (T) and 26.91% (G). The A + T content of protein coding genes and rRNA genes ranged from 59.65% (*rrn*S) to 68.63% (*nad3*) (Table 3), and the overall A + T content of the mt genome is 61.4%.

**Table 2:** **The organization of the mitochondrial genome of** ***Hypoderaeum conoideum***

**Gene/region**	**Positions**	**Size (bp)**	**Number of aa** ^**1**^	**Ini/Ter codons** ^**2**^	**Anticodons**	**In** ^**3**^
*cox*3	1-942	942	314	ATG/TAG		0
*trn*H	945-1011	67			GTG	+2
*cyt*b	1017-2126	1110	370	ATG/TAG		+5
*nad*4L	2132-2410	279	93	GTG/TAG		+5
*nad*4	2371-3654	1284	428	GTG/TAA		−40
*trn*Q	3662-3726	65			TTG	+7
*trn*F	3759-3824	66			TTG	+32
*trn*M	3837-3902	66			CAT	+12
*atp*6	3906-4424	519	173	ATG/TAG		+3
*nad*2	4428-5294	867	289	ATG/TAG		+3
*trn*V	5300-5367	68			TAC	+5
*trn*A	5391-5454	64			TGC	+23
*trn*D	5467-5532	66			GTC	+12
*nad*1	5533-6435	903	301	GTG/TAG		0
*trn*N	6443-6512	70			GTT	+7
*trn*P	6516-6581	66			AGG	+3
*trn*I	6583-6644	62			GAT	+1
*trn*K	6654-6721	68			TTT	+9
*nad*3	6726-7082	357	119	ATG/TAA		+4
*trn*S1	7087-7146	60			TCT	+4
*trn*W	7158-7225	68			TCA	+11
*cox*1	7229-8767	1539	513	GTG/TAG		+3
*trn*T	8797-8871	75			TGT	+29
*rrn*L^4^	8873-9851	979				+1
*trnC*	9852-9916	65			GCA	0
*rrn*S^4^	9917-10667	751				0
*cox*2	10668-11270	603	301	ATG/TAG		0
*nad*6	11302-11754	453	151	ATG/TAG		+31
*trn*Y	11755-11816	62			GTA	0
*trn*L1	11818-11883	66			TAG	+1
*trn*S2	11881-11945	65			TGA	−2
*trn*L2	11963-12025	63			TAA	+17
*trn*R	12029-12094	66			ACG	+3
*nad*5	12093-13658	1566	522	GTG/TAA		−1
*trn*G	13693-13757	65			TCC	+34
*trn*E	13764-13832	69			TTC	+6
Non coding region	13833-14180	348				0

The inferred length of amino acid sequence of 12 protein-coding genes: ^1^number of amino acids; ^2^initiation and termination codons; ^3^intergenic nucleotides; ^4^initiation or termination positions of ribosomal RNAs defined by adjacent gene boundaries.

**Table 3:** **Nucleotide contents of genes and the non-coding region within the mitochondrial genome of** ***Hypoderaeum conoideum***

**Gene**	**A (%)**	**G (%)**	**T (%)**	**C (%)**	**A + T (%)**
*cox*3	19.85	26.96	40.02	13.16	59.87
*cyt*b	18.83	23.87	44.86	12.43	63.69
*nad*4L	20.27	28.32	44.44	7.17	64.52
*nad*4	16.04	28.50	44.08	11.37	60.12
*atp*6	19.08	24.08	43.55	13.29	62.62
*nad*2	14.76	26.41	47.87	10.96	62.63
*nad*1	16.17	29.13	45.74	8.97	61.90
*nad*3	16.53	23.53	52.10	7.84	68.63
*cox*1	17.87	27.49	42.63	12.02	60.49
*rrn*L	23.90	26.46	36.06	13.59	59.96
*rrn*S	26.76	26.76	32.89	13.58	59.65
*cox*2	21.89	25.87	38.31	13.93	60.20
*nad*6	14.79	25.83	45.92	13.47	60.71
*nad*5	13.81	28.26	49.55	8.38	63.36
Non coding region	22.54	24.22	37.65	15.59	60.19

### Protein-coding genes

The *H. conoideum* mt genome has 12 protein-coding genes, including *nad*5, *cox*1, *nad*4, *cyt*b, *nad*1, *cox*3, *nad*2, *cox*2, *atp*6, *nad*6, *nad*3 and *nad*4L. For these protein coding genes, the initiation codon is ATG (seven of 12 protein genes), and GTG (five genes) (Table 2), which is in agreement with other digeneans [14,28]. The termination codon is TAG (seven of 12 protein genes) or TAA (five genes). The most frequently used codon is TTT (Phe), with the frequency of 7.96%, followed by GTT (Val: 5.99%), TGT (Cys: 4.63%), TTG (Leu: 4.30%) and TTA (Leu: 4.00%) (Table 4). The least used codons are GCC (Ala: 0.34%), CAC (His: 0.32%) and CGC (Arg: 0.11%).

**Table 4:** **Codon usage for 12 protein-coding genes in the mitochondrial genome of** ***Hypoderaeum conoideum***

**Codon**	**Amino acid**	**Number**	**Frequency (%)**	**Codon**	**Amino acid**	**Number**	**Frequency (%)**
TTT	Phe	315	8.88	ATT	Ile	130	3.66
TTC	Phe	46	1.30	ATC	Ile	21	0.59
TTA	Leu	149	4.20	ATA	Ile	58	1.63
TTG	Leu	292	8.23	ATG	Met	117	3.30
TCT	Ser	124	3.49	GTG	Met	117	3.30
TCC	Ser	21	0.59	ACT	Thr	46	1.30
TCA	Ser	21	0.59	ACC	Thr	11	0.31
TCG	Ser	36	1.01	ACA	Thr	16	0.45
TAT	Tyr	150	4.23	ACG	Thr	28	0.79
TAC	Tyr	21	0.59	AAU	Asn	58	1.63
TAA	Stop	3	0.08	AAC	Asn	8	0.23
TAG	Stop	9	0.25	AAA	Asn	25	0.70
TGT	Cys	105	2.96	AAG	Lys	52	1.47
TGC	Cys	13	0.37	AGT	Ser	75	2.11
TGA	Trp	34	0.96	AGC	Ser	15	0.42
TGG	Trp	78	2.20	AGA	Ser	25	0.70
CTT	Leu	65	1.83	AGG	Ser	65	1.83
CTC	Leu	4	0.11	GTT	Val	209	5.89
CTA	Leu	19	0.54	GTC	Val	21	0.59
CTG	Leu	43	1.21	GTA	Val	59	1.66
CCT	Pro	44	1.24	GCT	Ala	69	1.94
CCC	Pro	25	0.70	GCC	Ala	17	0.48
CCA	Pro	11	0.31	GCA	Ala	20	0.56
CCG	Pro	21	0.59	GCG	Ala	32	0.90
CAT	His	44	1.24	GAT	Asp	66	1.86
CAC	His	9	0.25	GAC	Asp	4	0.11
CAA	Gln	13	0.37	GAA	Glu	18	0.51
CAG	Gln	19	0.54	GAG	Glu	61	1.72
CGT	Arg	44	1.24	GGT	Gly	140	3.94
CGC	Arg	2	0.06	GGC	Gly	23	0.65
CGA	Arg	5	0.14	GGA	Gly	43	1.21
CGG	Arg	14	0.39	GGG	Gly	101	2.85

### Transfer RNA and ribosomal RNA genes, and non-coding regions

The *H. conoideum* mt genome encodes 22 tRNAs; all of them have a typical cloverleaf structure. The length of 22 tRNA genes ranges from 60 bp to 75 bp (Table 2). There are intergenic and overlapping nucleotides between adjacent tRNA genes (Table 2). The *rrn*S and *rrn*L are 751 bp and 979 bp in length, respectively (Table 2). The location of *rrn*S is between tRNA-Cys and *cox*2, and that of *rrn*L is between tRNA-Thr and tRNA-Cys, which is the same as other trematodes. In contrast to some other trematodes (two AT-rich regions), such as *F. hepatica* and *F. gigantica* [14,23], *O. felineus* [22] and *S. haematobium* [24], there is only one AT-rich region (348 bp) in the mt genome of *H. conoideum*, which is located between tRNA-Glu and *cox*3 (Figure 1 and Table 2), with an A + T content of 60.19% (Table 3).

### A comparison of nucleotide variability between *H. conoideum* and *F. hepatica*

**Figure 2:**
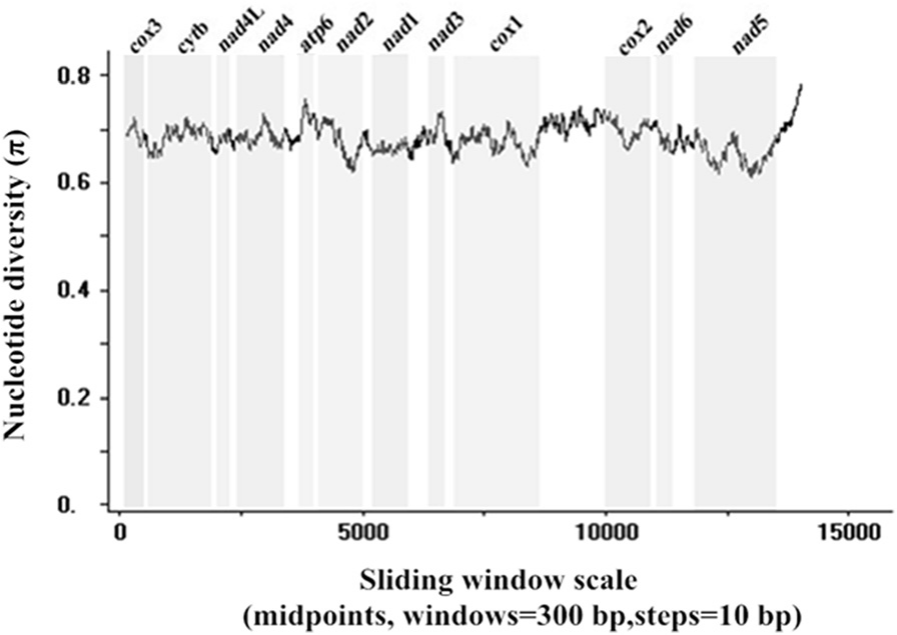
**Sliding window analysis of complete mt genome sequences of** ***Fasciola hepatica*** **and** ***Hypoderaeum conoideum*** **.** The black line indicates nucleotide diversity in a window of 300 bp (10 bp steps). Gene regions (grey) and boundaries are indicated.

A sliding window analysis of *H. conoideum* and *F. hepatica* using complete mt genomes showed the nucleotide diversity Pi (π) for 12 protein-coding genes (Figure 2). It indicated that the highest level of the mt sequence variability was within the gene *atp*6, and the lowest was within *nad*5. In our study, the most conserved protein-coding genes are *cox*1, *nad*2 and *nad*5, and the least conserved are *atp*6 and *nad*3.

### Phylogenetic relationships

**Figure 3:**
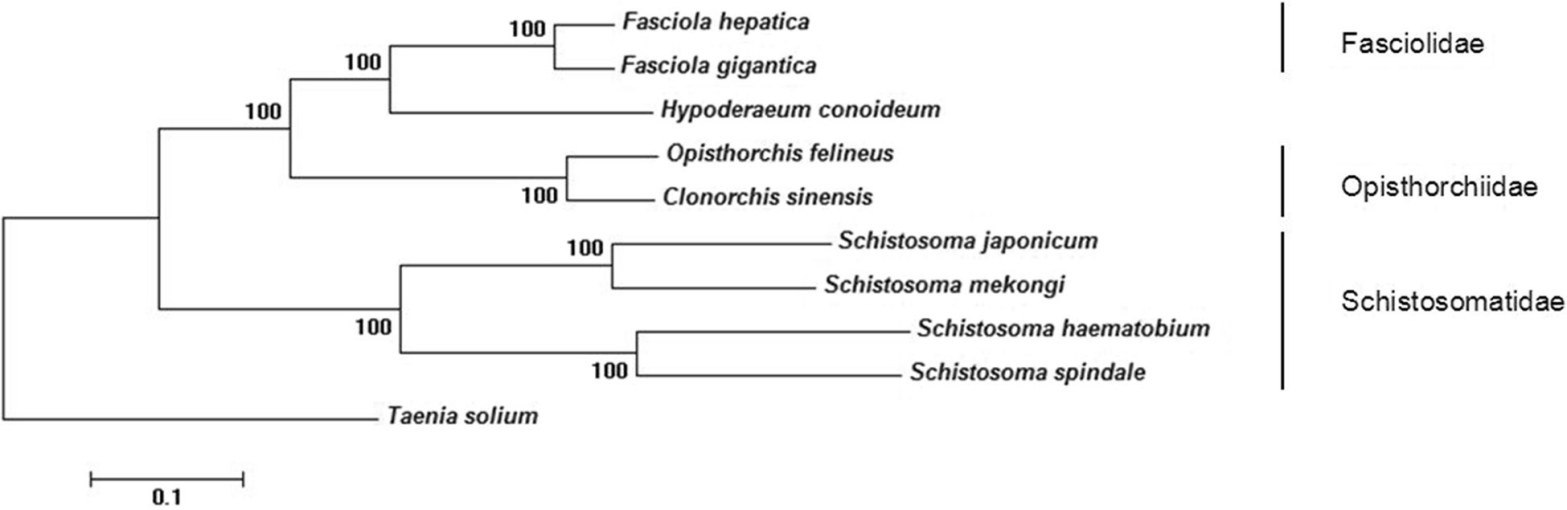
**Phylogenetic relationship of** ***Hypoderaeum conoideum*** **with selected trematodes; based on concatenated amino acid sequence data representing 12 protein-coding genes by neighbor-joining analysis, using** ***Taenia solium*** **as an outgroup.** Nodal support values are indicated (%); the bar indicates amino acid substitution per site.

We used concatenated amino acid sequence data representing 12 mt protein-coding genes of *H. conoideum*, eight other digeneans (*C. sinensis*, *F. gigantica*, *F. hepatica*, *O. felineus*, *S. haematobium*, *S. japonicum*, *S. mekongi* and *S. spindale*) and one tapeworm (*T. solium*) for a selective analysis of genetic relationships (Figure 3). The tree reveals two large clades with strong support (100%): one contains four members representing two families (Fasciolidae and Opisthorchiidae) and *H. conoideum*; the other clade contains four members of the Schistosomatidae. In the present analysis, *H. conoideum* had a relatively close genetic relationship with *F. hepatica* and other members of the Fasciolidae, followed by Opisthorchiidae, and then the Schistosomatidae. There was no difference in tree topology using the ML, MB and MP methods of analysis (not shown).

## Discussion

The present characterization of the mt genome of *H. conoideum* provides a basis for addressing questions regarding the biology, epidemiology and population genetics of *Hypoderaeum* spp. In addition, it will also assist in supporting taxonomic studies of *Hypoderaeum* spp. of other animals (e.g., chickens, ducks, geese and humans) as well as in tracking life cycles by identifying larval stages in different intermediate hosts using molecular tools.

Assisted by sliding window analysis, PCR primers could be selectively designed to regions conserved among different trematode species and flanking variable regions in the mt genome that are informative (based on sequencing from a small number of individuals from particular populations). PCR-coupled single-strand conformation polymorphism (SSCP) analysis [29] could then be employed to screen large numbers of individuals representing different populations and, based on such an analysis, samples representing all detectable genetic variability could be selected for subsequent sequencing and analyses. Such an approach has been applied to study the genetic make-up of the blood fluke *S. japonicum* from seven provinces in China [30,31].

Now that the *H. conoideum* mt genome is available, it would be interesting to undertake a comprehensive study of this morphospecies from various host species from different countries by integrating morphological data with PCR-based genetic analyses of adult worms and larval stages (from intermediate hosts) to begin to understand the epidemiology and ecology of *H. conoideum*. In addition to conducting targeted mt genetic analyses, it would also be useful to include analyses of sequence variability in the two internal transcribed spacers (ITS-1 and ITS-2), 18S and 28S of nuclear ribosomal DNA, because, for trematodes, these markers usually allow specific identification of trematodes. Importantly, although *H. conoideum* is recognized as a species, it is possible that cryptic species of this taxon might exist. This proposal could be tested using the mt markers defined here, together with ITS-1 and/or ITS-2.

## Conclusions

Our analysis showed that *H. conoideum* is genetically closely related to *F. hepatica* comparing with other trematodes. The mt genome of *H. conoideum* should be useful as a resource for comparative mt genomic studies of trematodes and DNA markers for systematic, population genetic and epidemiological studies of *H. conoideum* and congeners.

## References

[1] Sorensen RE, Curtis J, Minchella DJ (1998). Intraspecific variation in the rDNA its loci of 37-collar-spined echinostomes from North America: implications for sequence-based diagnoses and phylogenetics. J Parasitol.

[2] Steele JH (1982). CRC Handbook Series in Zoonoses. Section C: Parasitic Zoonoses (Vol. III).

[3] Yamaguti S (1958). Systema helminthum (Vol. I): part I. The digenetic trematodes of vertebrates.

[4] Rim HJ (1982). Echinostomiasis.

[5] Harinasuta T, Bunnag D, Radomyos P (1987). Intestinal fluke infections. Bailliere's clinical tropical medicine and communicable diseases.

[6] Yokogawa M, Harinasuta C, Charoenlarp P (1965). *Hypoderaeum conoideum* (Block, 1782) Diez, 1909, a common intestinal fluke of man in the north-east Thailand. Jpn J Parasitol.

[7] Nolan MJ, Cribb TH (2005). The use and implications of ribosomal DNA sequencing for the discrimination of digenean species. Adv Parasitol.

[8] Liu GH, Wang Y, Song HQ, Li MW, Ai L, Yu XL (2013). Characterization of the complete mitochondrial genome of *Spirocerca lupi:* sequence, gene organization and phylogenetic implications. Parasit Vectors.

[9] Hu M, Chilton NB, Gasser RB (2002). The mitochondrial genomes of the human hookworms, *Ancylostoma duodenale* and *Necator americanus* (Nematoda: Secernentea). Int J Parasitol.

[10] Saijuntha W, Sithithaworn P, Duenngai K, Kiatsopit N, Andrews RH, Petney TN (2011). Genetic variation and relationships of four species of medically important echinostomes (Trematoda: Echinostomatidae) in South-East Asia. Infect Genet Evol.

[11] Chai JY, Shin EH, Lee SH, Rim HJ (2009). Foodborne intestinal flukes in Southeast Asia. Korean J Parasitol.

[12] Wang GL (2013). Laboratory diagnostic techniques of parasites. Feeding Livestock.

[13] Tantrawatpan C, Saijuntha W, Sithithaworn P, Andrews RH, Petney TN (2013). Genetic differentiation of *Artyfechinostomum malayanum* and *A. sufrartyfex* (Trematoda: Echinostomatidae) based on internal transcribed spacer sequences. Parasitol Res.

[14] Le TH, Blair D, McManus DP (2001). Complete DNA sequence and gene organization of the mitochondrial genome of the liverfluke, *Fasciola hepatica* L. (Platyhelminthes; Trematoda). Parasitology.

[15] Hu M, Jex AR, Campbell BE, Gasser RB (2007). Long PCR amplification of the entire mitochondrial genome from individual helminths for direct sequencing. Nat Protoc.

[16] Thompson JD, Gibson TJ, Plewniak F, Jeanmougin F, Higgins DG (1997). The CLUSTAL_X windows interface: flexible strategies for multiple sequence alignment aided by quality analysis tools. Nucleic Acids Res.

[17] Ai L, Weng YB, Elsheikha HM, Zhao GH, Alasaad S, Chen JX (2011). Genetic diversity and relatedness of *Fasciola* spp. isolates from different hosts and geographic regions revealed by analysis of mitochondrial DNA sequences. Vet Parasitol.

[18] Le TH, Blair D, Agatsuma T, Humair PF, Campbell NJ, Iwagami M (2000). Phylogenies inferred from mitochondrial gene orders-a cautionary tale from the parasitic flatworms. Mol Biol Evol.

[19] Lowe TM, Eddy SR (1997). tRNAscan-SE: a program for improved detection of transfer RNA genes in genomic sequence. Nucleic Acids Res.

[20] Edgar RC (2004). MUSCLE: multiple sequence alignment with high accuracy and high throughput. Nucleic Acids Res.

[21] Librado P, Rozas J (2009). DnaSP v5: a software for comprehensive analysis of DNA polymorphism data. Bioinformatics.

[22] Liu GH, Gasser RB, Young ND, Song HQ, Ai L, Zhu XQ (2014). Complete mitochondrial genomes of the ‘intermediate form’ of Fasciola and Fasciola gigantica, and their comparison with F. hepatica. Parasit Vectors.

[23] Shekhovtsov SV, Katokhin AV, Kolchanov NA, Mordvinov VA (2010). The complete mitochondrial genomes of the liver flukes *Opisthorchis felineus* and *Clonorchis sinensis* (Trematoda). Parasitol Int.

[24] Littlewood DT, Lockyer AE, Webster BL, Johnston DA, Le TH (2006). The complete mitochondrial genomes of *Schistosoma haematobium* and *Schistosoma spindale* and the evolutionary history of mitochondrial genome changes among parasitic flatworms. Mol Phylogenet Evol.

[25] Nakao M, Sako Y, Ito A (2003). The mitochondrial genome of the tapeworm *Taenia solium*: a finding of the abbreviated stop codon U. J Parasitol.

[26] Yin F, Gasser RB, Li F, Bao M, Huang W, Zou F (2013). Genetic variability within and among *Haemonchus contortus* isolates from goats and sheep in China. Parasit Vectors.

[27] Mohandas N, Pozio E, La Rosa G, Korhonen PK, Young ND, Koehler AV (2014). Mitochondrial genomes of Trichinella species and genotypes-a basis for diagnosis, and systematic and epidemiological explorations. Int J Parasitol.

[28] Yan HB, Wang XY, Lou ZZ, Li L, Blair D, Yin H (2013). The mitochondrial genome of *Paramphistomum cervi* (Digenea), the first representative for the family Paramphistomidae. PLoS One.

[29] Gasser RB, Hu M, Chilton NB, Campbell BE, Jex AJ, Otranto D (2006). Single-strand conformation polymorphism (SSCP) for the analysis of genetic variation. Nat Protoc.

[30] Chilton NB, Bao-Zhen Q, Bøgh HO, Nansen P (1999). An electrophoretic comparison of *Schistosoma japonicum* (Trematoda) from different provinces in the People’s Republic of China suggests the existence of cryptic species. Parasitology.

[31] Zhu XQ, Bøgh HO, Gasser RB (1999). Dideoxy fingerprinting of low-level nucleotide variation in mitochondrial DNA of the human blood fluke, *Schistosoma japonicum*. Electrophoresis.

